# MRI-Based Quantitation of Hepatic Steatosis Does Not Predict Hypertrophy Rate after Portal Vein Embolization in Patients with Colorectal Liver Metastasis and Normal to Moderately Elevated Fat Fraction

**DOI:** 10.3390/jcm10092003

**Published:** 2021-05-07

**Authors:** Lea Hitpass, Iakovos Amygdalos, Paul Sieben, Vanessa Raaff, Sven Lang, Philipp Bruners, Christiane K. Kuhl, Alexandra Barabasch

**Affiliations:** 1 Department of Diagnostic and Interventional Radiology, RWTH Aachen University Hospital, Pauwelsstrasse 30, 52074 Aachen, Germany; psieben@ukaachen.de (P.S.); vraaff@ukaachen.de (V.R.); pbruners@ukaachen.de (P.B.); ckuhl@ukaachen.de (C.K.K.); abarabasch@ukaachen.de (A.B.); 2 Department of Surgery and Transplantation, RWTH Aachen University Hospital, Pauwelsstrasse 30, 52074 Aachen, Germany; iamygdalos@ukaachen.de (I.A.); svlang@ukaachen.de (S.L.)

**Keywords:** magnetic resonance imaging, liver, steatosis, embolization, hypertrophy

## Abstract

The aim of this study was to correlate the pre-procedural magnetic-resonance-imaging-based hepatic fat fraction (hFF) with the degree of hypertrophy after portal vein embolization (PVE) in patients with colorectal cancer liver metastases (CRCLM). Between 2011 November and 2020 February, 68 patients with CRCLM underwent magnetic resonance imaging (MRI; 1.5 Tesla) of the liver before PVE. Using T1w chemical shift imaging (DUAL FFE), the patients were categorized as having a normal (<5%) or an elevated (>5%) hFF. The correlation of hFF, age, gender, initial tumor mass, history of chemotherapy, degree of liver hypertrophy, and kinetic growth rate after PVE was investigated using multiple regression analysis and Spearman’s test. A normal hFF was found in 43/68 patients (63%), whereas 25/68 (37%) patients had an elevated hFF. The mean hypertrophy and kinetic growth rates in patients with normal vs. elevated hFF were 24 ± 31% vs. 28 ± 36% and 9 ± 9 % vs. 8 ± 10% (*p* > 0.05), respectively. Spearman’s test showed no correlation between hFF and the degree of hypertrophy (R = −0.04). Multivariable analysis showed no correlation between hFF, history of chemotherapy, age, baseline tumor burden, or laterality of primary colorectal cancer, and only a poor inverse correlation between age and kinetic growth rate after PVE. An elevated hFF in a pre-procedural MRI does not correlate with the hypertrophy rate after PVE and should therefore not be used as a contraindication to the procedure in patients with CRCLM.

## 1. Introduction

In up to 30% of patients diagnosed with colorectal cancer (CRC), liver metastases will occur within the first 5 years of diagnosis [1]. Liver resection remains the therapy of choice, as it is associated with a prolonged overall survival rate when complete resection is achieved [2,3]. However, in up to 60% of patients in whom surgical treatment is regarded as an option, an (extended) right hepatectomy—frequently accompanied by additional non-anatomic liver resections of the future liver remnant (FLR)—is needed to gain complete hepatic tumor eradication [4,5]. Without precautions, these patients are at significant risk of post-hepatectomy liver failure. To prevent this potentially life-threatening condition, patients with an FLR of less than 20% of the total liver volume (TLV) and otherwise healthy liver parenchyma and patients with an FLR of less than 40% of the TLV with underlying liver disease (e.g., severe steatosis, fibrosis or cirrhosis, or previous chemotherapy) require a presurgical induction of hypertrophy. Therefore, patients with an insufficient preoperative FLR, as determined by volumetry, routinely undergo portal vein embolization (PVE) to induce hypertrophy [6,7]. This minimally invasive procedure is performed percutaneously by a transhepatic puncture of the portal vein and an embolization of the right intrahepatic portal vein branches. In the two to four weeks after PVE, the average FLR volume increases by about 38% [8].

Many patients receive potentially hepatotoxic chemotherapy before undergoing PVE, which means they may already be suffering from an impaired liver function. More specifically, in up to 50% of patients receiving irinotecan and oxaliplatin-based regimens as neoadjuvant chemotherapy, chemotherapy-associated steato-hepatitis (CASH) has been reported in the histopathological examination of resected liver specimens [9]. Additionally, non-alcoholic fatty liver disease (NAFLD) is a rapidly emerging chronic liver disease and is also associated with an impaired liver function or even liver failure, suggesting the poor regenerative ability of the liver [10]. Therefore, patients with CASH and/or NAFLD can be expected to exhibit impaired FLR hypertrophy rates, which is why, currently, extended liver surgery and PVE is offered to these patients only after a careful and in-depth consideration of the risks and benefits in each individual case [11,12,13]. Although rates of major adverse events and technical failures leading to non-resectability after PVE are relatively low (approximately 0.1%, 0.4%, and 0.7%, respectively [8]), minor complications do occur quite frequently (in up to 40% of patients [8]).

All in all, there is a clear need for clinical predictors that can improve patient selection for patients with borderline liver tumor load requiring PVE for definitive surgery. The goal is to identify the patients who can expect a maximum benefit from this intervention and eventually proceed to surgery with a low risk of post-hepatectomy liver failure.

The aim of this study was therefore to investigate the role of the liver fat fraction, based on the pre-procedural magnetic resonance imaging (MRI), as a predictor of the success of PVE—i.e., by the degree of hypertrophy and the growth rates of the FLR—in patients with CRCLM.

## 2. Materials and Methods

Approval for this retrospective study was granted by the institutional review board (internal reference no EK 214/20). All patients provided written informed consent for interventional procedures. Decisions regarding oncological treatment, including the indication for PVE, were made by consensus in the institutional multidisciplinary tumor board, on which hepatobiliary surgeons, oncologists, radiotherapists, pathologists, and interventional body radiologists were present.

### 2.1. Patient Cohort

Patients who underwent PVE in preparation for a curative resection of hepatic colorectal liver metastases between November 2011 and February 2020 were retrospectively identified from the institutional database. Inclusion criteria for this study were (a) the availability of a contrast-enhanced pre-interventional in-house MRI acquired with a standardized protocol that enables the determination of hFF within a maximum of 1 month before the PVE procedure; (b) follow-up imaging (CT or MRI) to monitor the success of PVE—i.e., to assess the degree of hypertrophy—obtained just before surgery, or, in patients not undergoing surgery, within at least three weeks after PVE. The patients who did not proceed to resection were not excluded from the study, regardless of the underlying cause (e.g., extrahepatic or intrahepatic tumor progression or insufficient degree of hypertrophy of the FLR).

**Figure 1:**
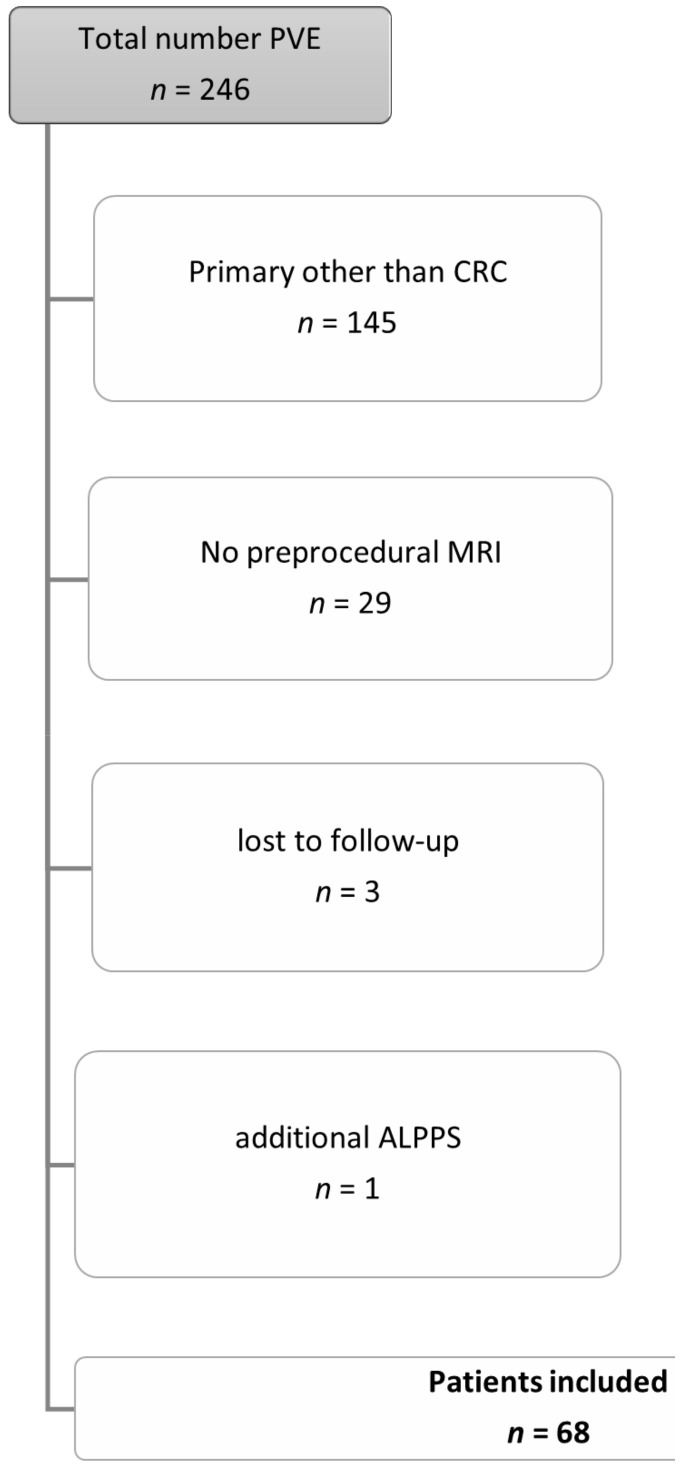
Inclusion and exclusion criteria for the patient cohort. Abbreviations: Portal vein embolization (PVE), colorectal cancer (CRC), magnetic resonance imaging (MRI), Associating Liver Partition with Portal Vein Ligation for Staged Hepatectomy (ALPPS).

Inclusion and exclusion criteria are shown in Figure 1.

### 2.2. Preprocedural MRI-Protocol

The MRI was performed according to a standardized protocol using a 1.5 Tesla MRI system (Achieva or Ingenia, Philips, Best, The Netherlands) with a 32-element body surface coil. The standardized protocol contained an axial T1w GRE (in-/opposed phase), axial diffusion weighted imaging, an axial and coronal T2w TSE with and without fat saturation, an axial T1w DCE, and an axial T1w GRE after contrast enhancement (THRIVE).

The detailed parameters of the pulse sequences used for further analysis in this study are described in Table 1.

### 2.3. Pre-/ and Postinterventional CT imaging

Computer tomography examinations were performed with a multislice multidetector CT scanner (Somatom Flash or AS 40, Siemens Medical Systems, Forchheim, Germany) at our department. Pre-interventional imaging generally included a chest and abdominal CT scan and a liver MRI, as part of oncological staging. The post interventional/ pre surgical imaging consisted at least of an abdominal CT scan.

CT scans of the abdomen were generated with a breath hold at the end of inspiration from the dome of the diaphragm to the proximal lower extremity. A non-ionic contrast agent (Ultravist 370; Bayer Schering Pharma AG, Berlin, Germany) was injected through an antecubital vein at a flow rate of 3.5 mL/s and a body weight adapted dose (1 mL per kg bodyweight) using an automatic injector.

Further technical details are shown in Table 2.

### 2.4. Portal Vein Embolization Procedure

All PVE procedures were carried out according to our departmental standards as follows: an ultrasound-guided, ipsilateral approach was taken for puncturing the right portal vein branch. A 6F sheath (Brite tip, Cordis, Hialeah, Florida) was placed into the main stem of the portal vein, followed by the insertion of a 5F catheter (SOS or Sidewinder). The embolization of all branches of the right portal vein took place in succession using a 4:1 mixture of n-butyl-2-cyanoacrylate (Histoacryl, Braun, Melsungen, Germany) and iodized oil (Lipiodol, Guerbet, Villepinte, France) through a 2.7 F-microcatheter (Renegate, Boston Scientific, Marlborough, Massachusetts). Subsequently, the puncture tract was embolized whilst the sheath and macro- and microcatheters were removed in succession.

### 2.5. Data Analysis

#### 2.5.1. Determining hFF

All quantitative MRI analyses were performed by a body radiologist (with 4 years of experience in abdominal MRI) who was blinded to the patients´ clinical data and outcomes.

For liver fat quantitation, regions of interest (ROI) with a diameter of 1.5–2 cm^2^ each were manually drawn in each segment of the left liver lobe on the T1w dual-GRE sequence (in-/opposed-phase; Dual-FFE), strictly avoiding vessels, focal liver lesions, and artefacts.

The liver fat fraction was calculated from the average of in- and opposed-phase signals using the formula below [14].

(1) $$\mathrm{Liver}\;\mathrm{fat}\;\mathrm{fraction}\;(\mathrm{FF})=\frac{IP-OP}{2\;IP}.$$

The patients were initially categorized according to their liver fat fraction into three groups: normal (<5% hFF), intermediately increased (5–30% hFF), and high (>30% hFF), according to the literature [15,16]. Since there were only two patients in the latter, all patients with an elevated liver fat fraction (hFF > 5%) were merged into one group. Negative values were set to zero.

#### 2.5.2. Determining the Hypertrophy Rate of the FLR

Volumetric liver analysis was performed using a state-of-the-art, semiautomatic liver analysis software (Intellispace Portal, Philips, Best, The Netherlands). The total liver volume, FLR volume, tumor volume (TV), FLR percentage, hypertrophy rate (HR) as well as kinetic growth rate (KGR) [17] were calculated using the pre- and postprocedural computed tomographies (CT) and the MRI using the formula below. The intrahepatic vessels, minor resections (before PVE or between PVE and the hypertrophy control) and the gallbladder were excluded from the liver volume.

**Figure 2:**
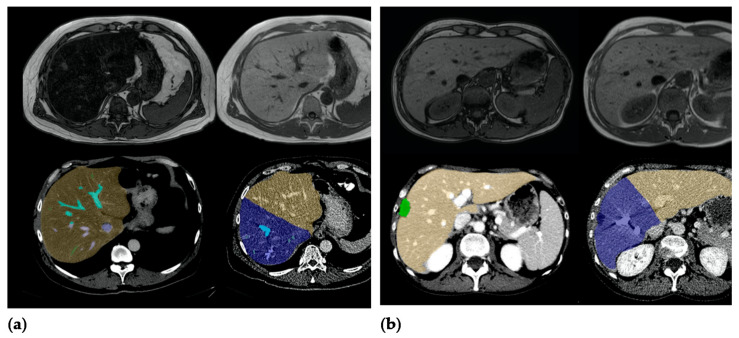
(**a**) Patient A, a 59-year-old male patient with steatosis (hFF 40.2%), hypertrophy rate 40.9%. (**b**) Patient B, a 47-year-old female patient without elevated hepatic fat fraction (hFF 4.9%), hypertrophy rate 9.3%.

The software output was corrected by a body radiologist (L.H., with 4 years of experience in body MRI and CT imaging), who corrected measurements on liver margins and added or subtracted the liver volume whenever needed (see Figure 2). To standardize the hypertrophy measurements, the FLR was always calculated according to a right hemihepatectomy by drawing the resection line along the right lateral margin of the middle hepatic vein.

Volumetric liver analysis was performed using CT images where available, since the majority of hypertrophy controls before surgery were carried out using CT, so this approach allowed for the best possible comparability. When no CT was available, MRI was used.

The KGR was calculated by dividing the degree of hypertrophy in the above-mentioned follow-up by the days elapsed since PVE. The degree of hypertrophy (%) was defined as the percentual increase in FLR volume between the pre- and post-PVE measurements. An impaired KGR was defined as a KGR of <2%/week according to the literature [17].

(2) $$\mathrm{FLR}\;(\%)=\frac{\mathrm{FLR}\left(\mathrm{mL}\right)}{\mathrm{TLV}\;\left(\mathrm{mL}\right)-\mathrm{TV}}\times 100.$$

### 2.6. Statistical Analysis

All continuous variables are shown as mean values ± standard deviation (SD). A multivariable regression analysis with six parameters (age, gender, FF, history of chemotherapy treatment, initial tumor mass, laterality of primary) was used to identify their impact on the hypertrophy rate after PVE. The Spearman correlation coefficient was used to assess the degree of monotonic association between the FLR hypertrophy (rate)/KGR and FF, chemotherapy treatment, initial tumor mass, and laterality of primary. All test results were analyzed in an explorative manner; thus, *p* values of *p* ≤ 0.05 were regarded as statistically significant. All statistical analyses were conducted using statistical software (SPSS, version 25; IBM, Armonk, NY, USA).

## 3. Results

### 3.1. Patient Cohort

In total, 101 patients with colorectal liver metastases underwent portal vein embolization in our department between November 2011 and February 2020. Of these, 68 patients (45 male, 23 females, mean age 60 ± 10 years) fulfilled the inclusion criteria for this study.

A total of 50/68 patients suffered from left-sided and a total of 18/68 patients from a right-sided colorectal cancer.

The mean tumor volume at baseline was 92 ± 216 mL (range: 0–1508 mL).

A total of 53/68 patients (78%) had received chemotherapy before undergoing PVE, 6 of whom continued their chemotherapy also in the time span between PVE and post-PVE hypertrophy control. Of the remaining 15/68 patients, three patients received chemotherapy in the time between PVE and surgery only, and twelve patients did not undergo chemotherapy at all.

Table 3 lists the type of chemotherapy regime received by the 53 patients.

The mean time span between PVE and imaging to assess the local hypertrophy of the FLR was 29 ± 22 d (range 11–131 d). The nine patients who underwent chemotherapy between PVE and the resection received follow-up imaging after a median time of 60 ± 42 days (range 13–131 d).

Four patients (5.8%) did not proceed to the resection due to significant intrahepatic tumor progression. Further details on patient characteristics are given in Table 3.

### 3.2. Volume of FLR

The mean FLR volume was 589 ± 193 mL (range: 127–1159 mL) before PVE and 730 ± 207 mL (range: 147–1378 mL) after a mean time interval of 29 days. The mean degree of hypertrophy was 25 ± 32%. The mean KGR was 9 ± 9% per week.

### 3.3. MRI Hepatic Fat Fraction

The MRI-derived mean fat fraction in our patient cohort was 6 ± 9% (range: 0 to 40%). A total of 43/68 (63%) patients had a fat fraction of less than 5% and were therefore categorized as having normal liver fat. Of the remaining 25 patients, 23 (92%) patients showed an intermediate liver fat level (hFF 5–30%) and 2/25 (8%) patients exhibited a severely elevated liver fat fraction (hFF > 30%).

#### Hepatic Fat Fraction Versus Degree and Rate of Hypertrophy

The mean degree of hypertrophy of the FLR in patients with a normal hFF was 24 ± 31% (range: −100 to 101%). The mean degree of hypertrophy of the FLR in patients with an elevated liver fat fraction was 28 ± 36% (range: −16 to 149 %). The mean growth rate per week was 9 ± 9% (range: −8 to 35%) in patients with a normal hFF, and 8 ± 10% (range: −4 to 38%) in patients with an elevated hFF.

**Figure 3:**
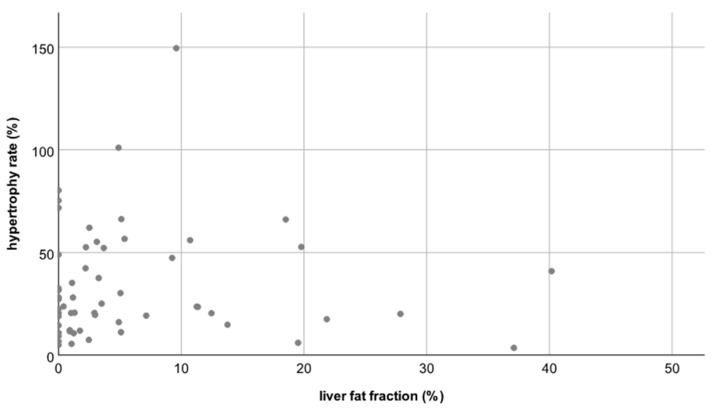
Scatterplot A: No correlation between the liver fat fraction (hFF) and the hypertrophy rate (HR); correlation coefficient R = −0.04.

**Figure 4:**
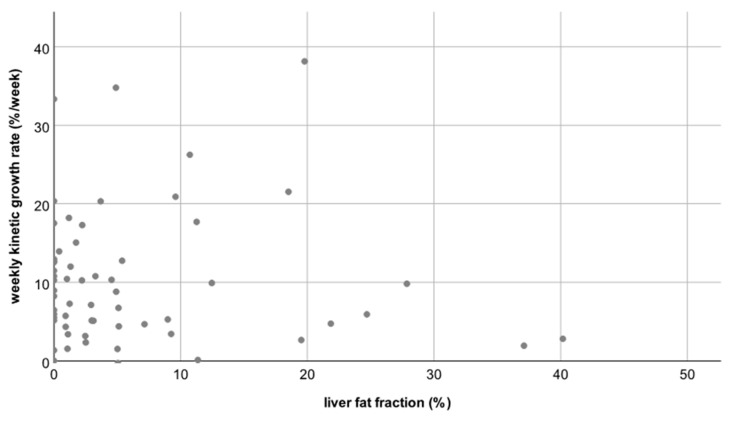
Scatterplot B: No correlation between the liver fat fraction (hFF) and the weekly kinetic growth rate; correlation coefficient R = −0.15.

Thus, neither the degree nor the rate of hypertrophy differed significantly between patients with a normal hFF and patients with an elevated hFF (*p* = 0.82 and *p* = 0.21, respectively, see Figure 3 and Figure 4).

### 3.4. History of Chemotherapy

In patients who had received or were currently undergoing chemotherapy, the mean degree of hypertrophy was 26 ± 36 % (range: −100 to 149%) at a mean growth rate per week of 9 ± 9 % (range: −8 to 38%). In patients without chemotherapy, the mean degree of hypertrophy was 22 ± 19 % (range: −9 to 57 %), at a mean growth rate per week of 9 ± 8 % (range: −3 to 26%). Thus, there was no statistically significant difference in the degree of hypertrophy or the hepatic growth rates between patients who did or did not undergo chemotherapy (*p* = 0.78 and *p* = 0.71, respectively).

### 3.5. Multivariate Analysis

**Figure 5:**
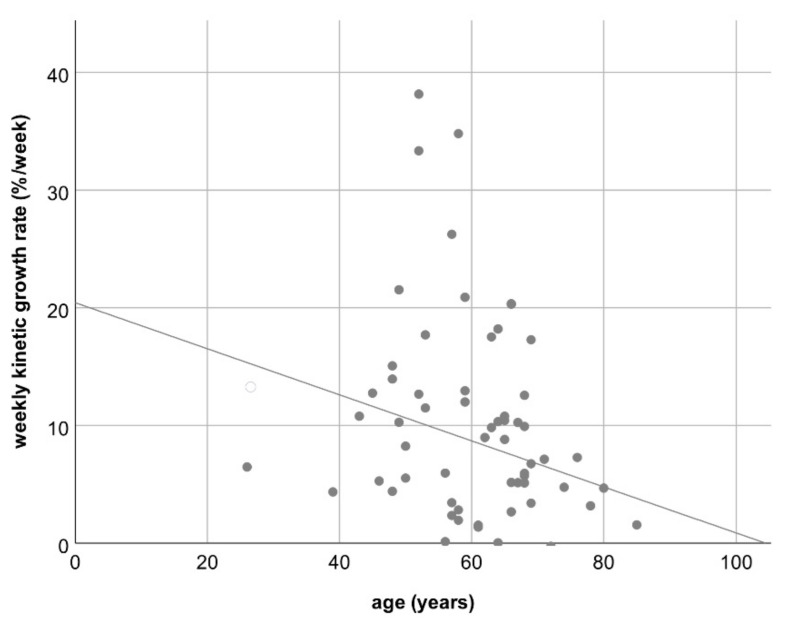
Scatterplot C: Loose inverse correlation between the patient’s age and the weekly kinetic growth rate; R = −0.24 (*p* = 0.04).

Multivariable analysis including gender, initial tumor mass, fat fraction, and laterality of primary colorectal cancer revealed no correlation between these factors and the degree of hypertrophy or the hepatic growth rate (Table 4 and Table 5), only a marginal inverse correlation between the patient’s age and the KGR/week (R = −0.24; *p* = 0.04; see Figure 5). No correlation was found for gender, initial tumor mass, fat fraction, and laterality of primary.

## 4. Discussion

The aim of this study was to investigate the association between the pre-procedural liver fat fraction and the success of portal vein embolization, as evidenced by the degree of hypertrophy observed in the future liver remnant (FLR). Our results show that although well over one third of patients (25/68 patients; 37%) had an abnormally elevated liver fat fraction based on MRI before the procedure, their degree of hypertrophy did not differ significantly from patients with a normal liver fat fraction (24 ± 31% vs. 28 ± 36%; *p* = 0.82).

Portal vein embolization is a standard procedure that helps to enable patients with borderline resectable CRLM to still undergo a potentially life-saving surgical procedure. Alternative procedures to PVE, such as ALPPS (Associating Liver Partition and Portal vein Ligation for Staged hepatectomy) or a combination of PVE with liver vein embolization, are associated with a significantly higher risk of major complications [18,19]. In comparison, PVE is a relatively safe procedure that is associated with a very low risk of major complications. However, after PVE, up to 5% of liver resections are cancelled due to insufficient FLR hypertrophy. Another 4% of patients die after hepatic resection, mainly due to hepatic failure most likely arising from small for size syndrome (SFSS) [8,20]. Hence, there is a significant clinical need to identify those patients that will successfully undergo FLR hypertrophy and liver surgery after PVE, and to identify the patients who should undergo alternative procedures, such as ALPPS, and/or patients who should be spared such procedures and should proceed with palliative chemotherapy right away.

An increased liver fat fraction is observed in patients with alcoholic or non-alcoholic fatty liver disease (AFLD, NACLD) and alcoholic and non-alcoholic steato-hepatitis (ASH, NASH). In the typical candidate for PVE, a frequent additional reason is chemotherapy-associated steato-hepatitis (CASH). Due to the increasing prevalence of alcoholic as well as non-alcoholic liver disease and due to the consistent use of chemotherapy prior to major liver surgery, an increased liver fat fraction is increasingly observed in PVE candidates.

The finding that there was no association between fat fraction and liver hypertrophy is somewhat counter-intuitive and against our expectation and contradicts a prior report by Barth et al. on 45 patients undergoing MRI prior to and after PVE [5]. However, this is in agreement with another study by Geisel et al. on 37 patients [21]. The discrepancy between our findings and those of Barth et al. might be attributable to the fact that in their study, a variety of surgical, interventional, and combined procedures were used for portal vein occlusion, including ALPPS, portal vein ligation, and percutaneous portal vein embolization. Our study, as well as that by Geisel et al., focused on patients undergoing percutaneous portal vein embolization.

Most patients who present with colorectal cancer liver metastases receive chemotherapy prior to surgery, usually containing either irinotecan or oxaliplatin (e.g., FOLFOX, FOLFIRI) or even both agents (FOLFOXIRI) according to standard oncologic guidelines (ESMO) [22]. In our cohort, over three-quarters of the cohort (78%) had received pre-interventional chemotherapy. Irinotecan is known to be associated with CASH, observed in up to 50% of patients [23]. However, oxaliplatin can also cause liver damage not due to steatosis, but due to sinusoidal obstruction (SOS) [24]. Accordingly, it is conceivable that the lack of association between the liver fat fraction and FLR hypertrophy could be caused by the fact that chemotherapeutic effects dominate the FLR growth kinetics, such that hypertrophy is reduced to a similar extent by either CASH or SOS. However, this interpretation cannot explain the observed lack of association in our study because only 8 of the 25 patients with an elevated liver fat fraction had irinotecan-induced CASH. Moreover, the hypertrophy rates in patients with or without chemotherapy did not differ significantly anyway—hence, it is unlikely that chemotherapy effects would override other factors that may modulate FLR growth rates. This was true even for the subset of patients who received both irinotecan and oxaliplatin in combination.

A loose inverse correlation was found between the patient’s age and the weekly KGR indicating that the elderly might have a reduced growth capacity of liver tissue. This seems plausible and in agreement with other studies that found that sarcopenia—as evidenced by a reduced psoas muscle mass—is associated with reduced hypertrophy rates of the FLR after PVE [25].

Our study has several limitations. First, our study cohort—although comparable to those of previously published studies on this issue—was still small. We strived to include a homogeneous patient cohort consisting of patients with colorectal liver metastases only undergoing a single type of portal vein occlusion procedure (in our case, PVE). Second, using chemical shift imaging for liver fat quantitation does not account for T2* relaxation effects. However, based on pathologic reports, no patient in our cohort showed signs of an abnormally high iron deposition in the liver. Hence, we do expect the T2* relaxation effects in our cohort to be only minor. Still, today there are newer and more precise MRI-based fat quantitation techniques, such as proton density fat fraction (PDFF) measurements, that justify investigation regarding their predictive value concerning FLR-growth.

Another limitation is that the follow-up scan time point was quite variable (mean 29 ± 22 d).

## 5. Conclusions

We can conclude that patients with liver metastases of colorectal cancer undergoing PVE do frequently (40%) exhibit an elevated liver fat fraction based on a pre-procedural MRI. However, a moderate elevation of liver fat fraction is not associated with the success of PVE. Therefore, an MRI-based liver fat quantitation cannot be used to select the patients who are suitable candidates for PVE/extended liver surgery; specifically, an elevated liver fat fraction should not be used to discourage PVE procedures in these patients.

## Figures and Tables

**Table 1:** Technical data MRI protocol.

Type of Scanner	1.5 T Achieva/Ingenia; Philipps Healthcare, Best, The Netherlands		
Pulse sequence type	2D T1w GRE (In- and opposed-phase)		2D T2w TSE
Orientation	axial		axial
Acquisition matrix	304		480
Field of view	380		380
Section thickness	6 mm		5 mm
TR	176		2200
TE	IP: 2.3	OP: 4.6	90
Breath compensation	Breath hold		Respiratory triggering

**Table 2:** Technical data CT protocol.

Type of Scanner	Somatom Flash, Siemens Medical System, Forchheim, Germany
Contrast agent	Non-ionic contrast agent (Ultravist 370, Bayer Schering Pharma AG, Berlin, Germany)
Dose of contrast agent	1 mL/kg body weight
Orientation	axial
Acquisition time	Arterial phase: 15 s Portal vein phase: 70 s Late phase: 300 s
Section thickness	5/1 mm 1/0.7 mm
Tube voltage	120 kV
Pitch factor	0.6
Section collimation	128 mm

**Table 3:** Patient demographics.

Patient Demographics of All 57 Patients	
Age, y (mean, SD)	60 ± 10
Gender (M, F)	45:23
Liver Tumor (number)	
Colorectal liver metastases	68
Left-sided primary	50
Right-sided primary	18
Initial overall tumor mass before PVE, mL (mean, SD)	80 ± 205
Pre-interventional chemotherapy (number)	53
FOLFOX	22
FOLFIRINOX	28
Capecitabine monotherapy	1
Unknown	2

**Table 4:** Correlation coefficient multivariable analysis.

Degree of Hypertrophy	Correlation Coefficient
Age	R = −0.20
Gender	R = 0.03
Liver fat fraction	R = −0.04
Chemotherapy treatment	R = 0.20
Initial tumor mass	R = 0.02
Laterality of primary CRC	R = −0.03

**Table 5:** Correlation coefficient multivariable analysis; ** *p* < 0.05, statistically significant.

Kinetic Growth Rate per Week	Correlation Coefficient
Age	R = −0.24 (*p* = 0.04) **
Gender	R = −0.19
Liver fat fraction	R = −0.15
Chemotherapy treatment	R = −0.07
Initial tumor mass	R = −0.08
Laterality of primary CRC	R = −0.07

## Data Availability

The data presented in this study are available on request from the corresponding author. The data are not publicly available due to data protection according to the institutional politics.
